# Acromegaly

**Published:** 1890-07

**Authors:** 


### Editorial, Etc.

ACROMEGALY.—At the Montefiore Home for Chronic Invalids in New York there is a patient suffering from a peculiar and incurable disease, the like of which is rarely met with, and never before in such a perfectly hopeless and completely developed state as in this instance. It is called acromegaly, but the name conveys no information of the horrible nature of the disease, which is one that baffles medical skill. The bones of the frame and cranium of a victim of acromegaly never cease to grow, but add bone tissue to bone tissue, cartilage to cartilage, until the individual is distended to an enormous size.

A Prussian woman, thirty-five years old, is afflicted with this disease and has been at the Montefiore Home for the past year. She was also at the German Hospital for fourteen months. The bones of both the frame and head have been steadily growing, the disease first developing when she was twenty-seven years old. No case of a similar nature, so complete in every detail, has before been met with by physicians either in this country or in Europe, though the disease has been observed before in a partly developed form. A case to which the public had their attention called was that of the "freak" at one time exhibited at the museums as the alleged big-footed girl.

The disease in its fully developed form is one that attacks the bones of the extremities first. The hands and feet begin to develope enormously. Then the extremities of each individual bone take on bone tissue and increase in size. At about the same time the bones of the cranium and the cartilages of the ears also develop. While this process of development in bone tissue is continuing an atrophy of muscular tissue is going on and certain glands that remain almost rudimentary in the healthy adult are developed. The medical fraternity is at a loss to account for this curious phenomenon.

The poor victim at the Montefiore Home now weighs 191 pounds, which is almost entirely due to the bone tissue. Her hands, feet and head are enormous. Her face is pale and her features are distorted. She suffers little pain from the unnatural disorder. Strange to say that with the growth of her bones the joints remain free and active and are as flexible as ever.

